# An association between pulmonary *Mycobacterium avium-intracellulare* complex infections and biomarkers of Th2-type inflammation

**DOI:** 10.1186/s12931-017-0579-9

**Published:** 2017-05-15

**Authors:** Paul E. Pfeffer, Susan Hopkins, Ian Cropley, David M. Lowe, Marc Lipman

**Affiliations:** 1 Royal Free London NHS Foundation Trust, London, UK; 2 William Harvey Research Institute, Queen Mary University of London, London, UK; 3 Institute of Immunity and Transplantation, Royal Free Campus, University College London, London, UK; 4 UCL Respiratory, Division of Medicine, University College London, Royal Free Campus, Pond Street, London, NW3 2QG UK

**Keywords:** Non-tuberculous mycobacteria, Mycobacterium avium-intracellulare, Eosinophil, Th2 lymphocyte

## Abstract

**Background:**

The rising incidence of pulmonary *Mycobacterium avium-intracellulare* complex (MAI) infection is unexplained but parallels the growing world-wide epidemic of allergic disease. We hypothesized an association between pulmonary MAI infection and Th2-type immune responses as seen in allergy.

**Methods:**

Biomarkers of patient Th2-type immune responses (peripheral blood eosinophil counts and serum IgE levels) were compared between patients with positive pulmonary samples for tuberculosis and non-tuberculous mycobacterial (NTM) infection. A further comparison of clinical characteristics, including respiratory co-morbidities, and biomarkers, was conducted between patients culturing MAI NTM and those culturing NTM other than MAI.

**Results:**

Patients culturing NTM from pulmonary samples had significantly higher peripheral blood eosinophil levels than those culturing *Mycobacterium tuberculosis.* Furthermore, patients culturing MAI compared to those culturing NTM other than MAI had higher eosinophil counts (mean 0.29x10^9^/L vs 0.15x10^9^/L, *p* = 0.010) and IgE levels (geometric mean 138kU/L vs 47kU/L, *p* = 0.021). However there was no significant difference in the frequency of asthma between the two NTM groups.

**Conclusions:**

There is an association between biomarkers of Th2-type immune responses and pulmonary MAI. Prospective and translational research could identify the direction of causation; and so determine whether our finding may be utilized within future management strategies for MAI.

## Background

The incidence of non-tuberculous mycobacterial (NTM) infection is rising [1], and this appears to be driven largely by an increase in pulmonary *Mycobacterium avium-intracellulare* complex (MAI) infection [2]. Pulmonary NTM disease is characterized by respiratory and constitutional symptoms, with significant impact on quality of life [3, 4]. Treatment requires long-courses of antibiotics, which often do not eradicate the mycobacteria [5]. Furthermore, many of the antimicrobials used to treat NTM disease have significant adverse-effects and drug-drug interactions [6]. As a result, often the benefit of attempting to treat localized infection is outweighed by its complications - leaving patients with persistent symptoms. Investigating the aetiology of pulmonary NTM infections may therefore enable us to devise more effective treatments and so manage the increasing number of affected individuals.

Whilst disseminated NTM infections are associated with severe immunodeficiency states such as HIV infection or defects in interferon gamma (IFNγ) and STAT3 pathways [7], the pathophysiology of localised pulmonary NTM infection is unclear [8]. Pulmonary NTM disease is most often seen in patients with chronic respiratory illness but this is perhaps confounded by doctors most often testing for NTM in such populations. Research in patients with cystic fibrosis demonstrates a specific association between NTM infection and allergic-bronchopulmonary aspergillosis (ABPA) [9] - suggesting that the increased predilection for NTM infections in patients with chronic lung disease is more complex than simply mycobacteria thriving in damaged lungs [10, 11].

In parallel with the increasing incidence of pulmonary NTM disease over the last decades, there is a globally rising prevalence of allergic diseases and the “atopic march” by which patients sequentially develop eczema, food allergies, allergic rhinitis and eosinophilic asthma [12, 13]. Dysregulated Th2 (T-helper lymphocyte type 2) immune responses are thought to underlie these states [14, 15]. Asthma itself is associated with increased susceptibility to, and severity of, certain infections, and this appears to be independent of the effect of corticosteroids [16]. For example, Kloepfer and colleagues have shown asthmatic children to be at greater risk of influenza infection [17], whilst Talbot and colleagues have demonstrated an association between asthma and invasive pneumococcal disease [18]. Furthermore, Th2-type inflammation is known to be associated with dissemination and impaired clearance of *Cryptococcus* [19, 20] and *Histoplasma* infections [21].

Following the observation in our NTM clinic of several patients with eosinophilia, we hypothesised that the increasing incidence of MAI disease reflects an increase in allergic disease, with a direct association between Th2-type cytokine predominant immune responses and MAI infection. Therefore, we reviewed biomarkers of Th2-type inflammation – peripheral blood eosinophil counts and serum Immunoglobulin E (IgE) levels [22] – in pulmonary NTM patients and compared them, where available, to a pulmonary tuberculosis cohort attending over the same period.

## Methods

### Patient Groups

A complete list of all patients with positive mycobacterial cultures from pulmonary samples at the Royal Free Hospital, London, UK, over the five years August 2010 – August 2015 was generated from the microbiology information system. Patients with known HIV, current haematological malignancy or significant primary immunodeficiency condition were excluded. Our NTM cohort was defined as those with at least two positive mycobacterial cultures, at least one identified NTM cultured and no positive cultures of *Mycobacterium tuberculosis* complex organisms. A cohort of patients with culture-positive pulmonary tuberculosis (TB) was similarly identified.

Patients’ pathology records were interrogated for the preceding eosinophil count (as an absolute value and as a percentage of the total white cell count) and total IgE before and closest to the date of the first positive mycobacterial culture. If these were not available then the first succeeding counts were recorded. A raised peripheral blood eosinophil count was defined as >0.4x10^9^/L and a raised serum IgE level as >150kU/L. An eosinophil count cut-off of 0.27x10^9^/L was also evaluated, given research showing this has a good sensitivity and specificity for detection of active eosinophilic airway inflammation in asthmatic patients [23].

Electronic records of clinical correspondence contemporaneous to the first positive NTM sample were also reviewed for evidence of patient or doctor reported co-morbid diagnoses such as asthma.

### Sample Analysis

Samples for mycobacterial analysis were received in sterile containers, homogenized and centrifuged as appropriate and decontaminated using NALC-NaOH method. All specimens were inoculated into growth indicators tubes (BD MGIT; BD, US), using 0.5 ml of processed specimen. Specimens that flagged positive on the BD MGIT system were examined for acid-fast bacilli and sub-cultured onto pH neutral pyruvate-based Löwenstein Jensen slope. For each patient with NTM the first two positive specimens were sent to the Public Health England (PHE) Mycobacterial Reference Laboratory for identification; thereafter new positive samples were sent monthly. The reference laboratory performed a Genotype Mycobacterium CM VER 2.0 analysis (Hain Lifescience, Germany).

Complete blood counts, including total white cell counts and eosinophil counts, were assayed using Sysmex XN-9000 analyzers (Sysmex, Japan). Total IgE measurement was performed on an ImmunoCAP 250 analyzer (ThermoScientific, US) using a fluorescence enzyme immunoassay technique (FEIA).

### Statistics

GraphPad Prism 6.0 (GraphPad Software, USA) was used for all parametric and non-parametric statistical tests. Unpaired t-tests were used to compare mean absolute eosinophil counts and mean percentage of total leukocyte counts between patient groups, and to compare IgE concentrations after logarithmic transformation of the variable. Chi-squared tests were used to compare distributions between patient groups of eosinophil counts in the ranges <0.27x10^9^/L vs 0.27-0.4x10^9^/L vs >0.4x10^9^/L. For comparisons of subject demographics between groups Chi-squared tests were employed (except for age in which an unpaired *t*-test was used). Figures show mean and standard deviation for all data except logarithmically transformed IgE concentrations for which geometric mean and 95% confidence interval are shown.

## Results

### Peripheral blood eosinophil counts are higher in patients with NTM infection than in patients with tuberculosis

Ninety-three patients cultured pulmonary NTM with at least two positive mycobacterial cultures over the five-year period. 19 were excluded from further analysis as they had known immunosuppressed states. Of the remaining 74 patients, 26 had two or more different species of NTM positively isolated in their cultures. Over the same period 55 patients had culture-positive pulmonary TB.

**Fig. 1:**
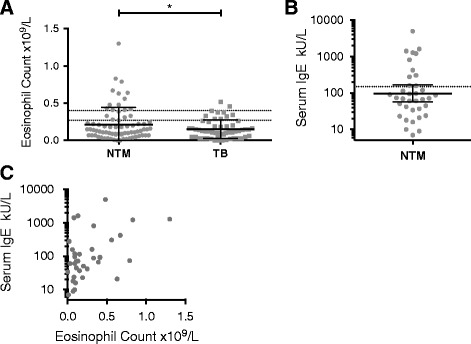
Peripheral blood eosinophil counts and serum IgE levels for patients culturing *Mycobacterium tuberculosis* and non-tuberculous mycobacteria. **a** Peripheral blood eosinophil counts of patients culturing non-tuberculous mycobacteria (NTM), *n* = 74, and *M tuberculosis* complex (TB), *n* = 55. *Dotted lines* at counts of 0.27x10^9^/L and 0.4x10^9^/L. Un-paired *t*-test with Welch’s correction; *, p ≤ 0.05. **b** Serum IgE levels for NTM patients, *n* = 36. *Dotted line* at 150kU/L. **c** Serum IgE levels plotted versus peripheral blood eosinophil counts for NTM patients

Peripheral blood eosinophil counts were significantly higher in the NTM cohort than in patients with pulmonary TB (mean 0.22x10^9^/L vs 0.15x10^9^/L, *p* = 0.049; Fig. 1a). 13 (18%) of NTM patients had an eosinophilia of >0.4x10^9^/L, with 19 (26%) a peripheral blood eosinophil count of ≥0.27x10^9^/L. This compared to only 2 (4%) of TB patients with an eosinophilia of >0.4x10^9^/L and 10 (18%) with a count ≥0.27x10^9^/L (*p* = 0.035, Chi-squared test).

Of the patients with NTM infection, 11 had a raised serum IgE >150kU/L out of 36 patients with a measured level (Fig. 1b). Although eosinophil and IgE levels were both elevated in some NTM patients (5 with eosinophil counts > 0.4x10^9^/L and total IgE >150 kU/L), others only had one or other raised (Fig. 1c). Serum IgE had only been assessed in one TB patient and therefore serum IgE levels could not be meaningfully compared between the TB patients and NTM patients.

### Prevalence of asthma is similar in patients culturing MAI complex mycobacterium and those culturing only other NTM

Given our hypothesis of an association between Th2 inflammation and MAI infection in particular, we next analyzed our NTM patient cohort by separating the patients into those culturing MAI complex mycobacteria as opposed to those culturing only NTM other than MAI complex mycobacteria. 36 (49%) individuals cultured MAI amongst their NTM isolates whilst in 38 only NTM other than MAI were identified. The ages of patients in both groups were not significantly different with mean (standard deviation) age of 63.3 years (16.43) for patients culturing MAI and 64.4 (15.36) for patients with NTM other than MAI. Similarly the gender distribution of patients was similar for both NTM groups (Table 1). The proportion of patients with different respiratory co-morbidities was comparable in people with MAI and those culturing only other NTM. In particular, proportions were similar for asthma (6 of 36 vs 4 of 38; *p* = 0.44, Chi-squared test) and COPD (9 of 36 vs 15 of 38; *p* = 0.18). However bronchiectasis was more common in the MAI group (15 of 36 vs 5 of 38; *p* = 0.006). Only one patient had a diagnosis of ABPA, and they had cultured MAI. Usage of inhaled corticosteroids was similar between the two groups (19 of 36 vs 15 of 38; *p* = 0.25).

**Table 1:** Demographics, respiratory co-morbidities and corticosteroid usage in NTM patients

	Patients with MAI complex NTM	Patients with NTM only other than MAI	
Total Subjects	36	38	
Mean Age/yrs (standard error of mean; range)	63.3 (2.74; 22.6 – 88.7)	64.4 (2.49; 31.3 – 90.3)	*p* = 0.78
Sex	22 female, 14 male	17 female, 21 male	*p* = 0.16
Subjects culturing multiple NTM species	18 (50%)	8 (21%)	*p* = 0.009
Asthma	6 (17%)	4 (11%)	*p* = 0.44
COPD	9 (25%)	15 (39%)	*p* = 0.18
Bronchiectasis	15 (42%)	5 (13%)	*p* = 0.006
ABPA	1 (3%)	0 (0%)	*p* = 0.30
Nasal Polyps	2 (6%)	1 (3%)	*p* = 0.52
on Inhaled Corticosteroids	19 (53%)	15 (39%)	*p* = 0.25
on Oral Corticosteroids	3 (8%)	3 (8%)	*p* = 0.94

Respiratory co-morbidities and usage of inhaled/oral corticosteroids in patients in our NTM cohort as detailed in medical notes contemporary to the dates of their first isolated NTM. Values represent number (%) except where specified

### Eosinophil counts and serum IgE levels are highest in patients culturing *Mycobacterium avium-intracellulare* (MAI) *complex* NTM

**Fig. 2:**
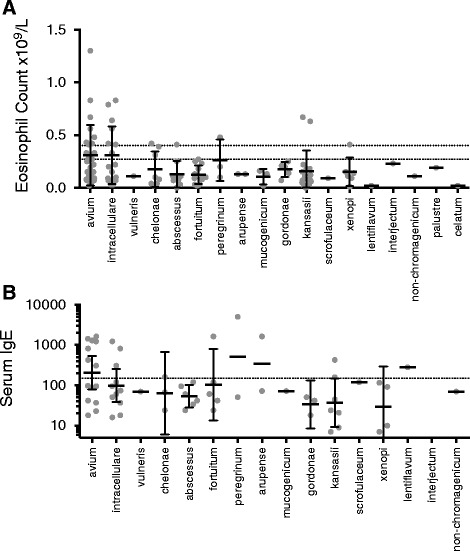
Eosinophil counts and serum IgE levels associated with different species of non-tuberculous mycobacteria. **a** Peripheral blood eosinophil counts and **b** serum IgE levels of patients culturing different species of NTM

We next addressed whether biomarkers of Th2 inflammation are particularly elevated in patients with MAI complex mycobacteria compared to other NTM. A high proportion of patients who had cultured *M avium* (8 of 26 patients) or *M intracellulare* (6 of 16) had an eosinophilia >0.4x10^9^/L (Fig. 2a). Similarly a high proportion of patients culturing either of these species of Mycobacterium had elevated IgE levels (Fig. 2b).

**Fig. 3:**
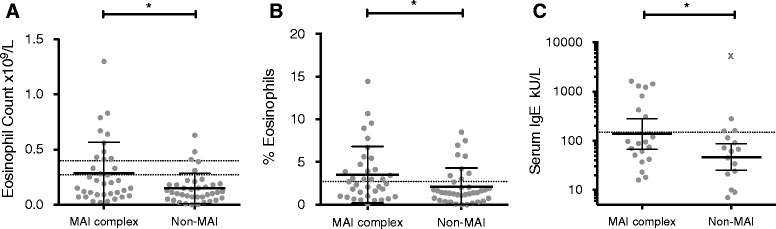
Eosinophil counts and serum IgE levels are significantly higher in patients culturing MAI complex mycobacterium than in patients only culturing other species of NTM. **a-c** Peripheral blood eosinophil counts, eosinophils as a percentage of the total leukocyte count, and serum IgE levels compared between patients whose samples cultured a *Mycobacterium avium-intracellulare* (MAI) complex NTM and those patients who cultured NTM only other than MAI mycobacteria. Unpaired t-tests with Welch’s correction (in the case of IgE levels after logarithmic transformation and exclusion of outlier indicated by symbol **X**); *, p ≤ 0.05

Peripheral blood eosinophils, either as an absolute count or as a percentage of the total leukocyte count, were significantly higher in patients culturing MAI complex compared to patients who had cultured NTM but no MAI organisms (mean 0.29x10^9^/L vs 0.15x10^9^/L, *p* = 0.010, and 3.5% vs 2.1%, *p* = 0.036; Fig. 3a-b).

After exclusion of an outlier with IgE >5000kU/L, serum IgE levels were significantly higher in individuals who had cultured MAI than in those only culturing other NTM (geometric mean 138kU/L vs 47kU/L, *p* = 0.021; Fig. 3c). Aspergillus specific IgE was measured in a small number of patients and could not be compared between NTM groups.

### Characteristics of eosinophilic patients with NTM infection

To examine whether there were particular characteristics associated with eosinophilic NTM infection, we examined this subgroup of 13 patients with peripheral blood eosinophil count >0.4x10^9^/L in further detail. The average age of these patients at the time of producing the NTM-cultured sample was 63 years. 8 were female and 5 male. 10 of the patients had cultured MAI complex NTM and 3 only other NTM. The mean IgE level was 948kU/L (measured in 9 patients). The 13 patients included the one patient with diagnosed ABPA and two further patients who had significantly raised Aspergillus specific IgE. All three cultured either *M. avium* or *M. intracellulare*. A further two had Aspergillus specific IgE within the normal range. 4 of the 13 eosinophilic patients had been diagnosed with asthma, including the 3 patients with the highest peripheral blood eosinophil counts – they had cultured either *M. avium* or *M. intracellulare* whereas the fourth asthmatic patient had cultured *M. xenopi*. All of the 4 asthmatic patients and 5 others were using inhaled corticosteroids.

CT thorax imaging was available for 12 of these 13 patients. Abnormalities on thoracic imaging were present in all of them. Tree-in-bud changes were evident in 3 patients and less defined/more widespread nodularity in a further 4. Airway changes on the bronchiectatic spectrum were present in 7. Upper lobe fibrosis with cavitation was found in 1 patient and a right upper lobe cavity evident in another.

## Discussion

Over recent decades there has been a rising incidence of both NTM infection, driven by increasing MAI infections, and atopic/allergic diseases, with the latter underpinned by Th2-type immune responses [1, 2, 12, 13]. We therefore hypothesized a possible association between MAI infection and Th2-type immune responses and investigated our cohort of patients with mycobacterial infection to evaluate this, using blood eosinophil count and serum IgE biomarkers of Th2-type responses. In the absence of a hematological malignancy, the Th2-type cytokine interleukin-5 (IL-5) is critical for the development of a peripheral eosinophilia [24] whilst B lymphocyte class-switching to IgE synthesis is dependent on the Th2-type cytokines IL-4/IL-13 [25]. We found significantly higher peripheral blood eosinophil counts in patients with NTM infection compared to pulmonary TB; and significantly higher eosinophil counts and serum IgE levels in patients culturing MAI complex NTM compared to those culturing only NTM other than MAI. Given the sample size in this study, the statistical significance of these differences is notable. We did not find asthma to be a more frequent co-morbidity in patients culturing MAI; however, not all cases of asthma are necessarily underpinned by allergic mechanisms or Th2-type inflammation [26]. Bronchiectasis was more common in patients culturing MAI NTM, and is of some interest given recent research regarding the association between NTM disease and bronchiectasis [27], and the frequency in asthma of airway changes along the bronchiectatic spectrum [28].

The association between MAI infection and Th2-type inflammation, as indicated by raised peripheral blood eosinophil levels and serum IgE levels, could have several explanations. One possibility is that inhaled corticosteroids used to treat Th2-associated airway inflammation (eg asthma, ABPA and eosinophilic COPD) may underlie the association. Previous studies have suggested steroids are a risk factor for NTM disease [9, 10]. If this were the explanation then it is of concern, as peripheral blood eosinophilia is increasingly regarded as a biomarker of under-treated steroid-responsive airways inflammation that requires increased steroid doses [23, 29] - which might paradoxically further increase the risk of NTM disease and impair anti-mycobacterial immune responses.

Alternatively, Th2-type inflammation itself could predispose to MAI infection. Successful host defense against mycobacteria with clearance/control of mycobacterial infection requires an effective Th1, and to lesser extent functioning Th17, immunological response rather than a Th2-type response [30, 31]. These different facets of the adaptive immune system are capable of cross-regulation – which allows an acute immune response to be specifically optimized to the character of any infection. However (chronic) disequilibrium between the different divisions of the adaptive immune system may lead to pathology and susceptibility to infection [32].

Mediators of Th2-type inflammation in allergic disease may suppress anti-mycobacterial responses in patients leading to persisting NTM infection. For example, the Th2-type cytokine IL-4 is known to inhibit Th1 and Th17 responses [33, 34]. Such mechanisms are thought to underlie the diminished IFN responses and more severe pathology seen in respiratory viral infections in asthmatics [35]. IL-5 also has the capacity to impair IFN responses [36]. If this were the case then suppressing Th2-type inflammation may be beneficial in MAI infection. For example, it is possible that anti-Th2 cytokine monoclonal antibody therapy could be a useful component of treatment for MAI, optimizing the body’s own anti-mycobacterial immune responses. It is notable that patients with common variable immune deficiency (CVID) and X-linked agammaglobulinaemia (XLA), who are unable to generate IgE, only rarely seem to develop NTM infections despite their increased susceptibility to other organisms and a very high incidence of bronchiectasis ([37, 38] and unpublished observations).

Finally, it is possible that MAI complex mycobacteria have developed defensive mechanisms to skew immune responses towards a Th2-type bias that decreases the ability of the immune system to clear the mycobacteria. For example, *Francisella tularensis* has previously been shown to promote macrophage differentiation into an ‘alternatively activated’ phenotype, more associated with allergic disease and tissue repair/remodeling than killing of intracellular pathogens [39].

There is a growing research field investigating the pulmonary microbiome in airway diseases: a prevailing theory is that loss of microbial diversity in patients with asthma may be responsible for dysregulated pulmonary immune tolerance and onset of disease pathology [40]. However, there is now increasing interest in the mechanisms by which respiratory microbes may perturb pulmonary immune responses and in particular how epithelial stimulation from microbial proteases may elicit Th2-type inflammation, with production of IL-4, IL-5 and IL-13 (for example) by other cells of the immune system such as innate lymphoid cells [41, 42].

Hence, there are several possible mechanisms by which MAI infection could directly promote aberrant pulmonary Th2-type inflammation. Interestingly, Fritscher and colleagues have reported a case-series of difficult-to-control asthma patients found to have NTM infection, the majority of whom had *Mycobacterium avium* complex infection [43]. In most of the patients treated with anti-mycobacterial therapy there was an improvement in their symptomatology.

A limitation of our work is that patient data were collected retrospectively. Therefore information on prevalence of eczema, food allergies and allergic rhinitis (the other diseases of the atopic march) was not available for most of these patients. Furthermore, the basis of asthma diagnoses in this cohort, whether patients had diagnostic lung function studies, and fractional exhaled nitric oxide (FeNO) measures of Th2-associated airway inflammation were not included in the available patient data. Additionally our eosinophilic patients were not universally screened for possible helminth infections that could potentially underlie the peripheral blood eosinophilia seen in these individuals (which is important given the current research interest into impaired anti-mycobacterial immune responses in patients with helminth and TB co-infections [44]). However the prevalence of helminth infection in London, UK, is very low and allergic/atopic disease is the cause of most peripheral blood eosinophilia in developed countries [45]. Further prospective research is needed to better investigate these observations, and on the basis of these results we would therefore advise that future studies of risk-factors for NTM disease should record whether patients have allergic/atopic co-morbidities, including aero-allergen testing, and include markers of Th2-type inflammation.

## Conclusions

We have shown an association between biomarkers of Th2-type immune responses and MAI complex NTM infection. It is possible that the developing epidemic of allergic/atopic disease may in part explain the increasing incidence of NTM infection – though the direction of causality remains undefined.

## Abbreviations

ABPA: Allergic broncho-pulmonary aspergillosis
COPD: Chronic obstructive pulmonary disease
IFN: Interferon
IgE: Immunoglobulin E
IL: Interleukin
MAI: *Mycobacterium avium-intracellulare* complex
NTM: Non-tuberculous mycobacteria
TB: *Mycobacterium tuberculosis*
Th: T-helper lymphocyte type-

## References

[1] Kendall BA, Winthrop KL (2013). Update on the epidemiology of pulmonary nontuberculous mycobacterial infections. Semin Respir Crit Care Med.

[2] Shah NM, Davidson JA, Anderson LF, Lalor MK, Kim J, Thomas HL (2016). Pulmonary Mycobacterium avium-intracellulare is the main driver of the rise in non- tuberculous mycobacteria incidence in England, Wales and Northern Ireland, 2007–2012. BMC Inf Dis.

[3] Mehta M, Marras TK (2011). Impaired health-related quality of life in pulmonary nontuberculous mycobacterial disease. Respir Med.

[4] Yeung MW, Khoo E, Brode SK, Jamieson FB, Kamiya H, Kwong JC (2016). Health-related quality of life, comorbidities and mortality in pulmonary nontuberculous mycobacterial infections: A systematic review. Respirology.

[5] Xu HB, Jiang RH, Li L (2014). Treatment outcomes for Mycobacterium avium complex: a systematic review and meta-analysis. Eur J Clin Microbiol Infect Dis.

[6] Griffith DE (1999). Risk-benefit assessment of therapies for Mycobacterium avium complex infections. Drug Saf.

[7] Wu U-I, Holland SM (2015). Host susceptibility to non-tuberculous mycobacterial infections. Lancet Infect Dis.

[8] Lake MA, Ambrose LR, Lipman MC, Lowe DM (2016). “Why me, why now?” Using clinical immunology and epidemiology to explain who gets nontuberculous mycobacterial infection. BMC Med.

[9] Mussaffi H, Rivlin J, Shalit I, Ephros M, Blau H (2005). Nontuberculous mycobacteria in cystic fibrosis associated with allergic bronchopulmonary aspergillosis and steroid therapy. Eur Respir J.

[10] Andrejak C, Nielsen R, Thomsen V, Duhaut P, Sorensen HT, Thomsen RW (2013). Chronic respiratory disease, inhaled corticosteroids and risk of non-tuberculous mycobacteriosis. Thorax.

[11] Sexton P, Harrison AC (2008). Susceptibility to nontuberculous mycobacterial lung disease. Eur Respir J.

[12] Eder W, Ege MJ, von Mutius E (2006). The asthma epidemic. N Engl J Med.

[13] Pearce N, Ait-Khaled N, Beasley R, Mallol J, Keil U, Mitchell E (2007). Worldwide trends in the prevalence of asthma symptoms: phase III of the International Study of Asthma and Allergies in Childhood (ISAAC). Thorax.

[14] Robinson DS (2010). The role of the T cell in asthma. J Allergy Clin Immunol.

[15] Durham SR, Till SJ, Corrigan CJ (2000). T lymphocytes in asthma: Bronchial versus peripheral responses. J Allergy Clin Immunol.

[16] Juhn YJ (2014). Risks for infection in patients with asthma (or other atopic conditions): is asthma more than a chronic airway disease?. J Allergy Clin Immunol.

[17] Kloepfer KM, Olenec JP, Lee WM, Liu G, Vrtis RF, Roberg KA (2012). Increased H1N1 infection rate in children with asthma. Am J Respir Crit Care Med.

[18] Talbot TR, Hartert TV, Mitchel E, Halasa NB, Arbogast PG, Poehling KA (2005). Asthma as a risk factor for invasive pneumococcal disease. N Engl J Med.

[19] Traynor TR, Kuziel WA, Toews GB, Huffnagle GB (2000). CCR2 Expression Determines T1 Versus T2 Polarization During Pulmonary Cryptococcus neoformans Infection. J Immunol.

[20] Pfeffer PE, Sen A, Das S, Sheaff M, Sivaramakrishnan A, Simcock DE (2010). Eosinophilia, meningitis and pulmonary nodules in a young woman. Thorax.

[21] Peng JK, Lin JS, Kung JT, Finkelman FD, Wu-Hsieh BA (2005). The combined effect of IL-4 and IL-10 suppresses the generation of, but does not change the polarity of, type-1 T cells in Histoplasma infection. Int Immunol.

[22] Westerhof GA, Korevaar DA, Amelink M, de Nijs SB, de Groot JC, Wang J (2015). Biomarkers to identify sputum eosinophilia in different adult asthma phenotypes. Eur Respir J.

[23] Wagener AH, de Nijs SB, Lutter R, Sousa AR, Weersink EJ, Bel EH (2015). External validation of blood eosinophils, FE(NO) and serum periostin as surrogates for sputum eosinophils in asthma. Thorax.

[24] Kouro T, Takatsu K (2009). IL-5- and eosinophil-mediated inflammation: from discovery to therapy. Int Immunol.

[25] Bacharier LB, Geha RS (2000). Molecular mechanisms of IgE regulation. J Allergy Clin Immunol.

[26] Woodruff PG, Modrek B, Choy DF, Jia G, Abbas AR, Ellwanger A (2009). T-helper type 2-driven inflammation defines major subphenotypes of asthma. Am J Respir Crit Care Med.

[27] Aksamit TR, O’Donnell AE, Barker A, Olivier KN, Winthrop KL, Daniels ML (2016). Adult Bronchiectasis Patients: A First Look at the United States Bronchiectasis Research Registry. Chest.

[28] Wang D, Luo J, Du W, Zhang LL, He LX, Liu CT (2016). A morphologic study of the airway structure abnormalities in patients with asthma by high-resolution computed tomography. J Thorac Dis.

[29] Pavord ID, Agusti A (2016). Blood eosinophil count: a biomarker of an important treatable trait in patients with airway disease. Eur Respir J.

[30] Lyadova IV, Panteleev AV (2015). Th1 and Th17 Cells in Tuberculosis: Protection, Pathology, and Biomarkers. Mediators Inflamm.

[31] Jasenosky LD, Scriba TJ, Hanekom WA, Goldfeld AE (2015). T cells and adaptive immunity to Mycobacterium tuberculosis in humans. Immunol Rev.

[32] Eberl G (2016). Immunity by equilibrium. Nat Rev Immunol.

[33] O’Garra A, Murphy K (1994). Role of cytokines in determining T-lymphocyte function. Curr Opin Immunol.

[34] Cohn L, Herrick C, Niu N, Homer RJ, Bottomly K (2001). IL-4 Promotes Airway Eosinophilia by Suppressing IFN-γ Production. Defining a Novel Role for IFN-γ in the Regulation of Allergic Airway Inflammation. J Immunol.

[35] Contoli M, Ito K, Padovani A, Poletti D, Marku B, Edwards MR (2015). Th2 cytokines impair innate immune responses to rhinovirus in respiratory epithelial cells. Allergy.

[36] Hatchwell L, Collison A, Girkin J, Parsons K, Li J, Zhang J (2015). Toll-like receptor 7 governs interferon and inflammatory responses to rhinovirus and is suppressed by IL-5-induced lung eosinophilia. Thorax.

[37] Winkelstein JA, Marino MC, Lederman HM, Jones SM, Sullivan K, Burks AW (2006). X-Linked Agammaglobulinemia. Report on a United States Registry of 201 Patients. Medicine (Baltimore).

[38] Oksenhendler E, Gérard L, Fieschi C, Malphettes M, Mouillot G, Jaussaud R (2008). Infections in 252 Patients with Common Variable Immunodeficiency. Clin Infect Dis.

[39] Shirey KA, Cole LE, Keegan AD, Vogel SN (2008). Francisella tularensis Live Vaccine Strain Induces Macrophage Alternative Activation as a Survival Mechanism. J Immunol.

[40] Parker W (2014). The “hygiene hypothesis” for allergic disease is a misnomer. BMJ.

[41] Tung HY, Landers C, Li E, Porter P, Kheradmand F, Corry DB (2016). Allergen-encoded signals that control allergic responses. Curr Opin Allergy Clin Immunol.

[42] Pfeffer PE, Corrigan CJ (2017). An Imbalance between Proteases and Endogenous Protease Inhibitors in Eosinophilic Airway Disease. Am J Respir Crit Care Med.

[43] Fritscher LG, Marras TK, Bradi AC, Fritscher CC, Balter MS, Chapman KR (2011). Nontuberculous mycobacterial infection as a cause of difficult-to-control asthma: a case–control study. Chest.

[44] Toulza F, Tsang L, Ottenhoff TH, Brown M, Dockrell HM (2016). Mycobacterium tuberculosis-specific CD4+ T-cell response is increased, and Treg cells decreased, in anthelmintic-treated patients with latent TB. Eur J Immunol.

[45] Lombardi C, Passalacqua G (2003). Eosinophilia and diseases: clinical revision of 1862 cases. Arch Intern Med.

